# Proteomic Changes during MCMV Infection Revealed by iTRAQ Quantitative Proteomic Analysis in Maize

**DOI:** 10.3390/ijms21010035

**Published:** 2019-12-19

**Authors:** Mingqing Dang, Qi Cheng, Ya Hu, Jianxiang Wu, Xueping Zhou, Yajuan Qian

**Affiliations:** 1 State Key Laboratory of Rice Biology, Institute of Biotechnology, Zhejiang University, Hangzhou 310058, China; dangmingqing2008@163.com (M.D.); 21716069@zju.edu.cn (Q.C.); 21616132@zju.edu.cn (Y.H.); wujx@zju.edu.cn (J.W.); zzhou@zju.edu.cn (X.Z.); 2 State Key Laboratory for Biology of Plant Diseases and Insect Pests, Institute of Plant Protection, Chinese Academy of Agricultural Sciences, Beijing 100193, China

**Keywords:** maize chlorotic mottle virus, iTRAQ, photosynthesis, disulfide isomerases, peroxiredoxin, VIGS

## Abstract

Maize chlorotic mottle virus (MCMV) has been occurring frequently worldwide and causes severe yield losses in maize (*Zea mays*). To better investigate the destructive effects of MCMV infection on maize plants, isobaric tagging for relative and absolute quantitation (iTRAQ)-based comparative proteomic analysis was performed on MCMV infected maize cv. B73. A total of 972 differentially abundant proteins (DAPs), including 661 proteins with increased abundance and 311 proteins with reduced abundance, were identified in response to MCMV infection. Functional annotations of DAPs and measurement of photosynthetic activity revealed that photosynthesis was decreased, while the abundance of ribosomal proteins, proteins related to stress responses, oxidation-reduction and redox homeostasis was altered significantly during MCMV infection. Two DAPs, disulfide isomerases like protein ZmPDIL-1 and peroxiredoxin family protein ZmPrx5, were further analyzed for their roles during MCMV infection through cucumber mosaic virus-based virus-induced gene silencing (CMV-VIGS). The accumulation of MCMV was suppressed in ZmPDIL-1-silenced or ZmPrx5-silenced B73 maize, suggesting ZmPDIL-1 and ZmPrx5 might enhance host susceptibility to MCMV infection.

## 1. Introduction

Plants are generally exposed to diverse biotic and abiotic stresses that significantly affects plant growth and development. In order to defend against pathogen attacks, plants trigger overlapping sets of defense responses including activation of secondary metabolites and hormone signaling, reducing photosynthesis, accumulation of reactive oxygen species and callose, reinforcement of the cell walls, and rearrangement of cytoskeleton [1,2,3,4,5,6,7]. Extensive studies of comparative transcriptional and proteomic analysis revealed the roles of transcriptional reprogramming of defense genes and posttranslational modification of proteins in plant immunity to pathogens [2,8,9,10].

Maize is an important staple food and energy crop worldwide. However, the quality as well as production of maize are always threatened by pathogen invasion. Several RNA viruses—such as sugarcane mosaic virus (SCMV), rice black streaked dwarf (RBSDV), maize dwarf mosaic virus (MDMV), maize rough dwarf virus (MRDV), and maize chlorotic mottle virus (MCMV)—have been reported as common viral agents infecting maize [11,12,13]. Synergistic interactions of MCMV with potyviruses, such as wheat streak mosaic rymovirus or SCMV, are fairly common in maize fields [13,14].

MCMV, which comprises a linear, positive sense, single-stranded RNA of 4436 nucleotides (nt), is the only member in the genus *Machlomovirus*, family *Tombusviridae*. Two subgenomic RNAs (sgRNAs), named as sgRNA1 and sgRNA2, are transcribed during the viral infection process [15]. The first open reading frame (ORF) in the MCMV genomic RNA (gRNA) encodes a P32 protein unique to MCMV. A former study showed MCMV accumulation and the severity of viral symptoms were dramatically decreased in the absence of P32 [16]. The P50 protein and its stop codon readthrough protein, P111 (the ratio of P50 to P111 is still unclear), are related to highly conserved RNA dependent RNA polymerases (RdRps) encoded by other members in the family *Tombusviridae* [15,16]. In the 3’-terminal third of the genome, several ORFs are expressed from sgRNA1 [17]. The first ORF in sgRNA1 encodes the P7a and a P7a stop codon readthrough product known as the P31 protein. A small ORF after the P7a ORF, known as P7b, was identified recently [16]. P7a and P7b proteins are closely related to the movement protein 1 (MP1) of viruses in the family *Tombusviridae* or MP2 encoded by panicoviruses [18,19], which are required for MCMV cell-to-cell movement. The P31 protein facilitates virus systemic infection in plants [16]. The second AUG codon on sgRNA1 encodes the MCMV coat protein (CP) [17]. A 337 nt sgRNA2 was also found in MCMV-infected protoplasts and plants [17].

Through RNA-Seq, many differentially expressed genes were identified to associate with plant pathogen infection in maize [20,21,22,23,24]. For example, gene expression profile analysis revealed that the brassinosteroid (BR) pathway was significantly altered after MCMV-infected maize plants [22]. However, posttranslational modifications usually modulate gene expression and protein accumulation, the transcriptional levels do not correlate well with the protein abundances. Therefore, the analysis of differential protein profile might be a more efficient way to accurately discover the key factors participating in plant immunity to pathogens. Proteomic analysis of maize–virus interactions have addressed the effect of SCMV, RBSDV, or MDMV on maize protein abundance [11,25,26,27]. However, to our knowledge, scarcely any proteomic datasets in response to MCMV infection have been reported. To effectively determine the molecular mechanism(s) underlying MCMV infection, we used the isobaric tags for relative and absolute quantification (iTRAQ)-based comparative proteomic approach to analyze maize cv. B73 plants infected with MCMV. The results of the present study provide a detailed whole-proteome information on the effects of MCMV infection in maize.

## 2. Results

### 2.1. Phenotype Shown on MCMV-Infected Maize Plants

**Figure 1:**
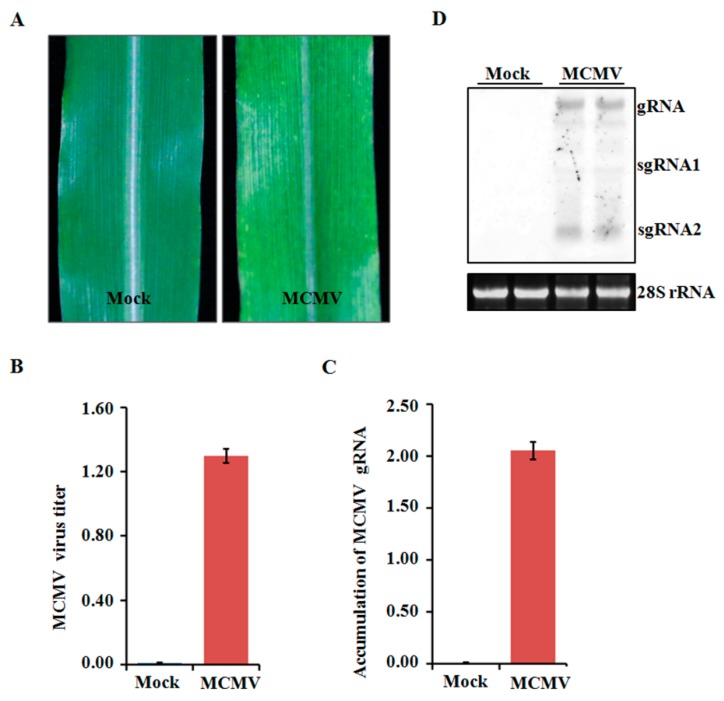
Assays of maize cv B73 plants inoculated with MCMV. (**A**) Typical symptom induced by MCMV infection. The leaves were photographed at 11 dpi. (**B**) Detection of MCMV accumulation by ELISA using an anti-MCMV monoclonal antibody. (**C**,**D**) Detection of MCMV gRNA accumulations by qRT-PCR using MCMV specific primers or northern blot using MCMV specific probe. Bars indicate the means ± standard deviations (SD) from three independent experiments.

Four-leaf stage maize plants were inoculated with crude extracts from MCMV-infected leaf tissues and periodically monitored for disease symptom. All the inoculated seedlings developed mosaic symptoms in their young leaves at 11 dpi (Figure 1A). To determine the accumulated levels of MCMV in systemically infected leaves, tissues were collected and tested using ELISA. The MCMV titer was markedly higher in the MCMV-infected samples than that in those mock-inoculated control plants (Figure 1B). Further confirmation was obtained using qRT-PCR and northern blot, which showed that the accumulation levels of MCMV gRNA were remarkably increased in the MCMV-infected samples compared with those controlled samples (Figure 1C,D).

### 2.2. iTRAQ-Based Proteomic Analysis

**Figure 2:**
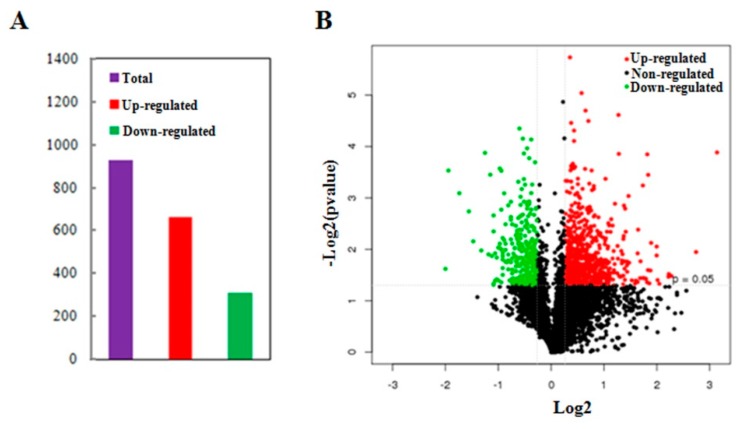
Summary of differentially abundant proteins (DAPs) responsive to MCMV infection. (**A**) The total number of DAPs containing the increased abundance and reduced abundance. The numbers above the bars are the exact numbers of DAPs. (**B**) Volcano plot showing distribution of the total identified protein. The *x*-axis shows various fold change groups and the *y*-axis shows the *p* value.

In order to identify the differentially abundant proteins responsive to MCMV infection, total proteins were extracted from the MCMV-infected or mock-inoculated maize seedlings and detected using comparative iTRAQ analysis. About 333,899 spectra were obtained between the two groups and 73,966 of them were matched to known spectra using the Mascot analysis software. From the 60,212 unique spectra obtained, 13,606 peptides were identified and 4546 unique proteins were detected at 95% confidence level. Furthermore, among the unique proteins, about 47% of them had >10% sequence coverage, and over 52% of them matched at least two peptides, allowing to quantify their abundance more precisely. Further analysis of the 4546 quantified proteins, a total of 972 proteins were identified as differentially abundant proteins (DAPs) with significant changes (*p*-value < 0.05, cut-off point fixed at >1.2 or <0.83), including 661 proteins with increased abundance and 311 proteins with reduced abundance (Figure 2A, Tables S1 and S2). Additionally, ‘volcano plot’ showed the distribution of DAPs mainly located between 1.2-fold to 2.0-fold change while only about 12.6% of the DAPs changed by more than 2-fold (Figure 2B).

### 2.3. Impacts of MCMV Infection on the Maize Global Proteome

**Figure 3:**
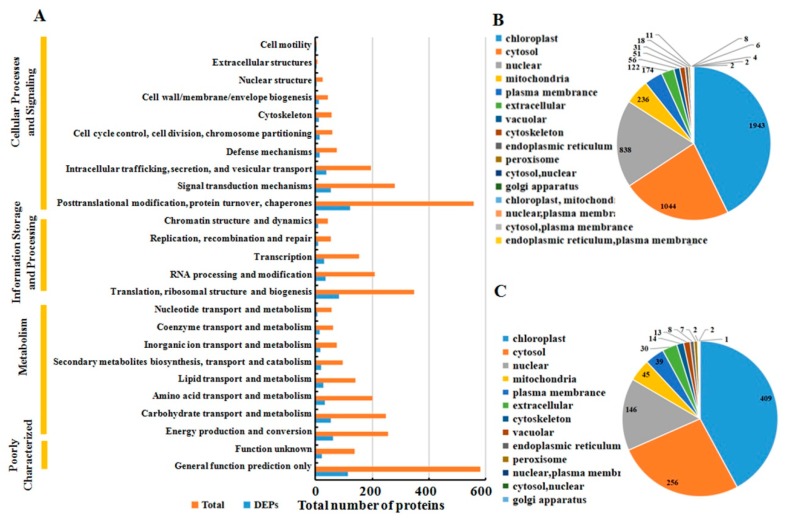
Classification of all identified proteins and DAPs. (**A**) all identified protein (orange) and DAPs (blue) are divided into different terms based on KOG analysis. Subcellular location of all identified proteins (**B**) and DAPs (**C**).

The functions of all the identified proteins and the DAPs after MCMV infection were classified using the KOG database annotation. The results illustrated in Figure 3A revealed that 1300 identified proteins and 276 DAPs were involved in ‘cellular process and signaling’, and 1132 identified proteins and 233 DAPs were involved in ‘metabolism’. Additionally, the category of ‘information storage and processing’ included 807 identified proteins and 167 DAPs, respectively.

All identified proteins and DAPs were further assorted according to their subcellular locations (Figure 3B). For identified proteins, 16 different subcellular components were found, including 1943 chloroplast-localized proteins, 1044 cytosol-localized proteins, 838 nuclear-localized proteins and 236 mitochondria-localized proteins. For DAPs, only 13 different subcellular components were found, including 409 chloroplast-localized proteins, 256 cytosol-localized proteins, 146 nuclear-localized proteins, and 45 mitochondria-localized proteins.

### 2.4. GO and KEGG Analysis of DAPs under MCMV Infection

**Figure 4:**
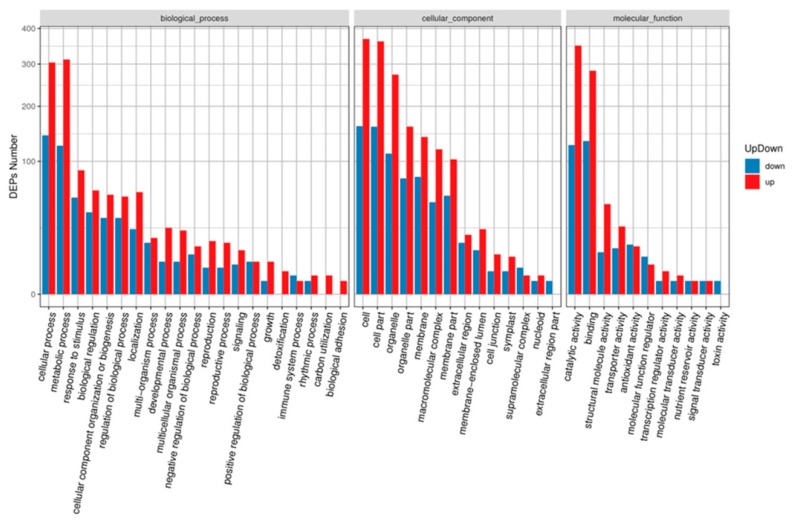
GO terms analysis of the DAPs identified during MCMV infection. The *x*-axis indicate the grouped GO terms containing cellular component, molecular function, and biological process. The *y*-axis shows cluster frequencies of the GO terms.

Gene Ontology (GO) annotation showed that all DAPs were grouped into three different GO categories, containing 16 terms in the cellular component category, 11 terms in the molecular function category, and 21 terms in the biological process category (Figure 4). Among these terms, cellular process (447 DAPs), metabolic process (437 DAPs), response to stimulus (140 DAPs), biological regulation (99 DAPs), cellular component organization or biogenesis (89 DAPs), regulation of biological process (87 DAPs), and localization (83 DAPs) were the top five terms in the biological process category. Cell (529 DAPs), cell part (521 DAPs), and organelle (385 DAPs) terms in the cellular component category; and catalytic activity (476 DAPs) and binding activity (416 DAPs) terms in the molecular function category were enriched with DAPs.

**Figure 5:**
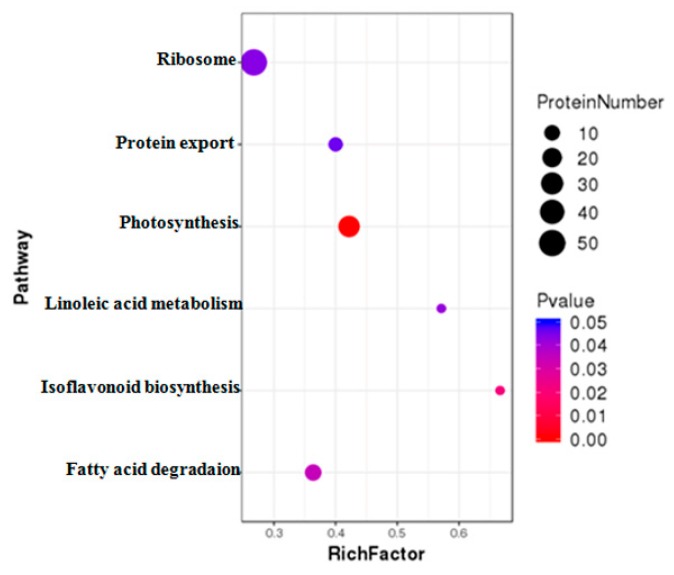
KEGG enrichment analysis of the DAPs identified during MCMV infection. The *y*-axis indicate the significantly enriched KEGG terms. The *x*-axis shows cluster rich factor of the KEGG terms.

DAPs were further classified using Kyoto Encyclopedia of Genes and Genomes (KEGG) pathway annotation and enrichment analysis. The results revealed that those DAPs under MCMV infection could be divided into 115 different pathways. Among these pathways, six pathways were significantly enriched with DAPs, including ribosome, photosynthesis, protein export, fatty acid degradation, linoleic acid metabolism, and isoflavonoid biogenesis related terms (Figure 5).

### 2.5. Photosynthetic Activity Decreased during MCMV Infection

Viral infection usually triggers dramatic changes in the expression of photosynthesis-related proteins, which are important in plant–virus interactions [28]. Decreased photosynthesis, mostly at the proteome level, is usually associated with viral symptoms. Additionally, some photosynthesis-related proteins have been reported to participate in plant defense against viruses [28]. PSI and PSII core subunits and the oxygen evolving proteins are common targets for virus-induced changes [28,29]. Consistent with previous studies, our iTRAQ data and KEGG pathway annotation identified that 14 DAPs associated with photosynthesis were reduced abundance and 13 DAPs related to photosynthesis were increased abundance responsive to MCMV infection. Among these DAPs, eight PSII-associated proteins were identified as significantly modulated proteins while two oxygen-envolving enhancer proteins constituted by PsbP (GRMZM2G047954_P01, photosystem II oxygen-evolving enhancer protein 2) and PsbQ (GRMZM5G804323_P01, photosystem II oxygen-evolving enhancer protein 3) were found to downregulate 1.41-fold and 1.89-fold, respectively. The decrease in abundance of PetE (GRMZM2G071450_P01, plastocyanin) and PetF (GRMZM2G043162_P01, ferredoxin) that involve in photosynthetic electron transport was 2.1- and 1.37-fold compared to the controlled maize plants.

**Figure 6:**
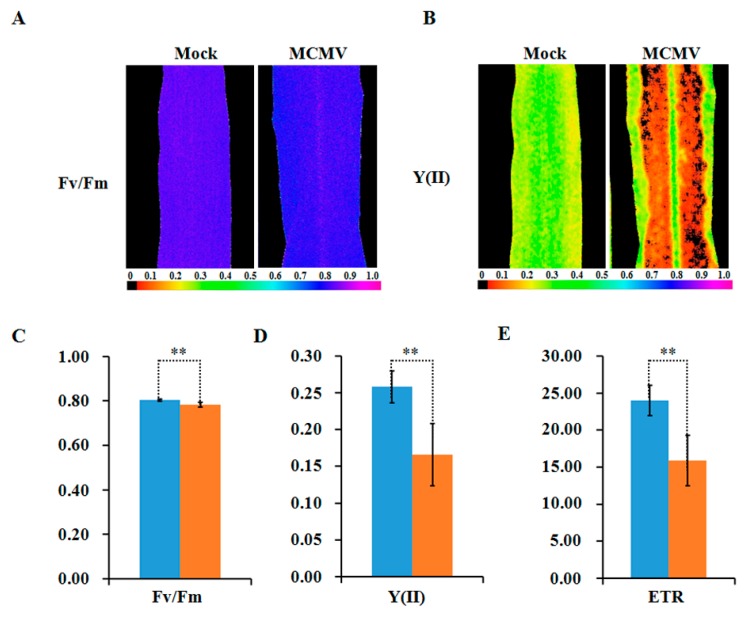
Detection of photosynthetic activity after MCMV infection. (**A**,**B**) Images showing the maximum photochemical efficiency (Fv/Fm) and the actual photochemical efficiency (Y(II)) of PSII of whole leaves. The color code depicted at the bottom of the image ranges from 0 (black) to 1.0 (purple). (**C**) Average Fv/Fm values; (**D**) average Y(II) values; and (**E**) average ETR values of whole leaves are shown. The blue denote mock-inoculated plants and the orange denote MCMV-infected plants. The bars represent the mean values, with standard errors of the mean, for one representative experiment (*n* = 10). *, statistically different at *p* < 0.05 level.

To further reveal the decreased abundance in these photosynthesis-related proteins interference in plant photosynthesis, the chlorophyll fluorescence parameters were measured in MCMV-infecting or controlled maize leaves. As shown in Figure 6A–E, the maximum photochemical efficiency of PSII (Fv/Fm), the actual photochemical efficiency of PSII (Y(II)), and the electron transfer rate (ETR) exhibited marked decreases after MCMV infection.

### 2.6. Alteration of Ribosomal Protein Abundance during MCMV Infection

Ribosomes are the cellular machinery needed for protein synthesis, which comprise a large (60S or 50S) subunit, a small (40S or 30S) subunit, rRNAs and other proteins. In eukaryotic cells, ribosomes are found in the cytoplasm, mitochondria, and in plant chloroplasts [30,31]. In the present study, the abundance of ribosomal proteins was altered significantly in MCMV-infected samples compared to the mock-inoculated control, as revealed by KEGG annotation and enrichment analysis. A total of 50 DAPs were annotated as ribosome-related pathways, and 40 out of them were significantly upregulated, suggesting a remarkable increase abundance in ribosomal protein levels (Table S3). Among these modulated proteins, 19 DAPs were annotated as 60S ribosomal proteins, 13 DAPs were discovered to be associated with 40S ribosomal proteins and 10 DAPs were related to mitochondrial/chloroplast ribosomal proteins. The top hit of these DAPs in reduced abundance was GRMZM5G851698_P02 (annotated as 40S ribosomal protein S6) that was downregulated by 3.85-fold while the top hit of these increased abundant DAPs was GRMZM2G152573_P04 (annotated as 60s ribosomal protein L34) and GRMZM2G044800_P02 (annotated as 40S ribosomal protein S11) that were upregulated by 2.53-fold.

### 2.7. Enrichment of Stress Response-Related Proteins during MCMV Infection

Plants have evolved many defense strategies to survive under abiotic and biotic stress conditions. We found that a total of 30 DAPs were annotated to be involved in defense response and response to biotic stimulus in our GO annotation result, including 17 proteins with increased abundance and 13 proteins with reduced abundance (Table S4). Among these DAPS, heat shock protein 70 (GRMZM2G428391_P01) and WD repeat-containing protein domain phosphoinositide-interacting protein 3 (GRMZM2G122607_P01) were remarkably accumulated, while ribosome-inactivating protein 2 (GRMZM2G119705_P01) and calcium-dependent protein kinases (GRMZM2G112057_P01) were markedly downregulated in response to MCMV infection. On the other hand, it was notable that the GRMZM2G156861_P01 (annotated as lipoxygenase, LOX) was upregulated by 1.28-fold while the level of GRMZM2G040095_P02 (annotated as lipoxygenase 6, LOX6) was downregulated by 1.25-fold in MCMV-infected plants compared with mock-inoculated plants, suggesting that functional segregation among different LOXs upon MCMV infection.

### 2.8. Differential Regulation of Redox Homeostasis Related Proteins

Plants defense abiotic and biotic stresses through regulating the production of reactive oxygen species (ROS) and the antioxidant machinery [32]. Interestingly, we found that the levels of several oxidation-reduction process-related proteins were significantly altered in MCMV-infected plants compared to the control plants (Table S5), indicating the crucial functions of antioxidation during MCMV infection. For example, the levels of 2-oxoglutarate dehydrogenase E1 (GRMZM2G151041_P01), known to recruit its E2 and E3 components to generate ROS, was increased in abundance with more than 2-fold after MCMV infection, while the level of monooxygenase (GRMZM2G089121_P02) was upregulated by 1.91-fold. Isocitrate dehydrogenase is revealed to be an important enzyme catalyzing the reduction of H_2_O_2_, and in our results, isocitrate dehydrogenase 1 (GRMZM2G432128_P01) was increased in abundance with 1.88-fold. Meanwhile, a total of 20 DAPs related to cell redox homeostasis were identified through GO annotation (Table S6). Among these DAPs, protein disulfide isomerase 1 (ZmPDIL-1, GRMZM2G091481_P01) were remarkably accumulated with 1.55-fold increased abundance while peroxiredoxin-5 (ZmPrx5, GRMZM2G036921_P01) was downregulated by 1.24-fold, indicating these proteins might be important host factors involving in regulating plant resistance or tolerance to virus infection stress.

### 2.9. Transcriptional Level Analysis for Selected DAPs

**Figure 7:**
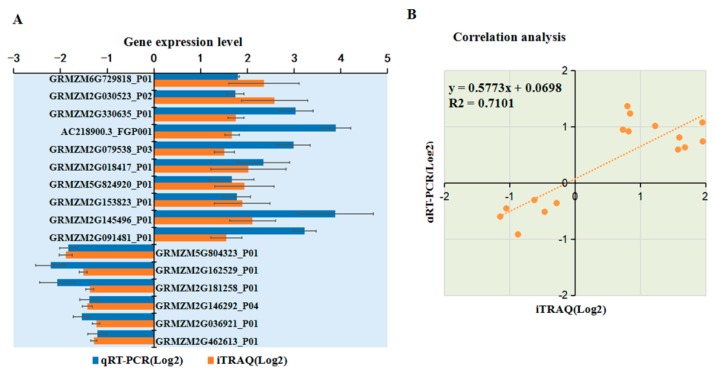
Transcriptional analyses of selected DAPs. (**A**) Transcriptional analyses of selected DAPs. The *y*-axis shows the 16 selected DAPs for the analyses. The *x*-axis indicates the relative expression levels. The bars represent the mean values, with standard errors of the mean, for one representative experiment (*n* = 6). (**B**) Correlation analysis of selected DAPs btween iTRAQ data and qRT-PCR analysis. The *x*-axis and *y*-axis are shown in the Log2 scale.

To verify the changes in protein levels determined using iTRAQ analysis, 18 DAPs were selected and analyzed using qRT-PCR. Among 11 upregulated proteins identified from the iTRAQ comparative analysis, the mRNA expression levels of 10 DAPs were upregulated after MCMV infection according to qRT-PCR analysis (Figure 7A). Among seven downregulated proteins identified from the iTRAQ analysis, six were significantly downregulated at the transcriptional level after MCMV infection (Figure 7A). As shown in Fig 7B, the trend of the changes in the levels of these DAPs determined by qRT-PCR and iTRAQ analyses matched, although the value of the changes identified by the two methods did not match exactly. In addition, several proteins, such as GRMZM2G005984_P01 and GRMZM2G104384_P01 showed inconsistent results between the iTRAQ and qRT-PCR analyses. There is a large amount of evidence that transcript and protein levels do not match and our result is yet another example of this. Post-transcriptional regulation or post-translational modifications might be responsible for these differences.

### 2.10. Functional Analyses of Two Selected DAPs through Virus-Induced Gene Silencing

**Figure 8:**
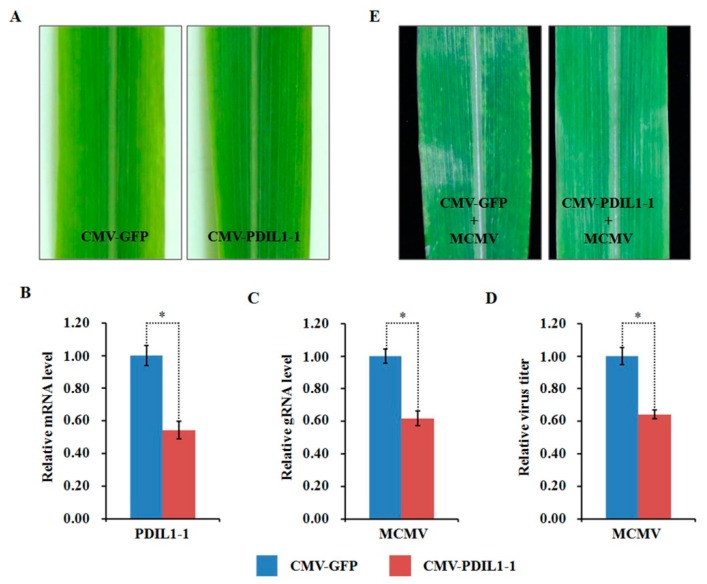
Effects of silencing ZmPDIL-1 expression through CMV-based VIGS on MCMV infection in maize. (**A**) Typical symptoms in the ZmPDIL-1-silenced B73 leaves were similar to that in the non-silenced control leaves. (**B**) qRT-PCR analysis of ZmPDIL-1 expression in ZmPDIL-1-silenced and control B73 plants agroinoculated with either TRV:ZmPDIL-1 or TRV:GFP. The error bars showed standard deviations. (**C**,**D**) Silencing of ZmPDIL-1 suppressed MCMV accumulation in systemic infected leaves. The gene silencing efficiency and viral gRNA accumulations were detected by qRT-PCR (**C**) and viral titer was determined by ELISA (**D**). Bars indicated the means ± standard deviations (SD) from three independent experiments; *, statistically different at *p* < 0.05 level; the results were determined by the Student’s *t*-test. (**E**) Typical symptoms in the ZmPDIL-1-silenced or non-silenced B73 leaves inoculated with MCMV at 11 dpi.

**Figure 9:**
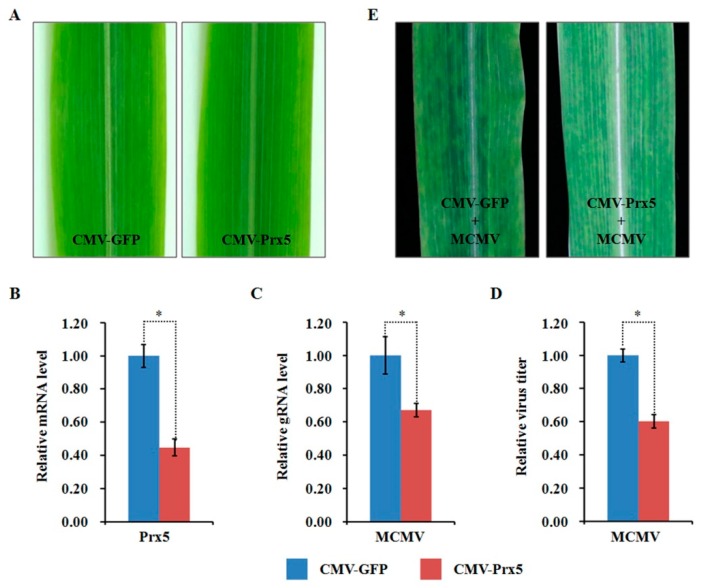
Effects of silencing ZmPrx5 expression through CMV-based VIGS on MCMV infection in maize. (**A**) Typical symptoms in the ZmPrx5-silenced B73 leaves and the non-silenced control leaves. (**B**) qRT-PCR analysis of ZmPrx5 expression in ZmPrx5-silenced and control B73 plants agroinoculated with either TRV:ZmPrx5 or TRV:GFP. The error bars showed standard deviations; (**C**,**D**) Silencing ZmPrx5 expression impaired MCMV accumulation in systemic infected leaves. The viral gRNA accumulations were detected by qRT-PCR (**C**) and viral titers was determined by ELISA (**D**). Bars indicated the means ± standard deviations (SD) from three independent experiments; *, statistically different at *p* < 0.05 level; the results were determined by the Student’s *t*-test. (**E**) Typical symptoms in the ZmPrx5-silenced or non-silenced B73 leaves inoculated with MCMV at 11 dpi.

Compared to the mock-inoculated plants, the abundance of ZmPDIL-1 was significantly upregulated while the abundant of ZmPrx5 was remarkably downregulated in MCMV infected maize plants. However, the differential abundance of proteins, whether up- or downregulated, does not absolutely indicate antiviral activity. Hence, we investigated the potential function(s) of the regulated proteins during MCMV infection by silencing candidate ZmPDIL-1 and ZmPrx5 in maize using a CMV-based VIGS system for genetic validation. The silencing efficiency of the target genes ZmPDIL-1 and ZmPrx5 was detected in young systemic leaves that did not cause obvious phenotypic changes at 15 dpi (Figure 8A and Figure 9A), based on qRT-PCR analyses. The results revealed that the ZmPDIL-1 relative transcript level in the CMV-ZmPDIL-1-inoculated plants was reduced to approximately 54%, the relative transcript level of ZmPrx5 in the CMV- ZmPrx5-inoculated plants was reduced to about 45% of the value obtained from the controlled plants (Figure 8B and Figure 9B). Then, the leaves of ZmPDIL-1 and ZmPrx5-silenced B73 plants were challenged with MCMV. ELISA and qRT-PCR were used separately to analyze the accumulation of gRNA and the virus titer of MCMV in these inoculated plants. The qRT-PCR results showed that MCMV gRNA levels in the ZmPDIL-1-silenced and ZmPrx5-silenced plants were decreased to approximately 62% and 67% compared with those in the non-silenced at 11 dpi (Figure 8C). The MCMV titer in the ZmPDIL-1-silenced plants were decreased to approximately 62% and 60%, respectively, compared with those in the control plants (Figure 8D and Figure 9D). Consistent with the reduced MCMV accumulation in the ZmPDIL-1 and ZmPrx5-silenced plants, disease symptoms in these plants were also alleviated (Figure 8E and Figure 9E). All these results indicated that the abundances of ZmPDIL-1 and ZmPrx5 might play crucial roles in maize defense again MCMV infection.

## 3. Discussion

MCMV infection has resulted in destructive losses in maize production and has rapidly spread into many regions worldwide [33]. At all growth stages, most current commercial maize cultivars are susceptible to MCMV and no extremely resistant maize cultivars have been identified [34]. Thus, there is an urgent need to determine the molecular mechanisms about how MCMV establish an infection and how the plant reacts to viral infection. Herein, an iTRAQ-based comparative proteomic approach was performed in the present study to provide the whole-proteome information during MCMV infection of maize.

### 3.1. Photosynthesis Decreased during MCMV Infection

The most common viral symptom of plant virus infection is caused by chlorosis and the subsequent loss of chlorophyll, which usually imply the occurrence of chloroplast-virus interactions. In plants, the chloroplast is not only a common target of plant virus but also the organelle that conducts photosynthesis [28]. Previous researches have indicated that decreased photosynthesis, mostly at the proteome level, is a common phenomenon in plants infected by numerous viruses including tobacco mosaic virus, cucumber mosaic virus, plum pox virus and peanut stunt virus [35,36,37,38]. A research showed seven photosynthesis-related proteins were significantly downregulated and the photosynthetic activity was strongly suppressed in the systemic leaves during SCMV infection [26]. Consistent with these previous findings, our iTRAQ-based proteomic analysis and measurement of photosynthetic rates both indicated maize photosynthesis was severely impaired during MCMV infection. A variety of reports suggested photosynthesis-related proteins could play active roles in plant defense viruses. Kong and associates confirmed that silencing of host Psbp, an oxygen-involving enhancer protein of PSII located in chloroplast, in both rice and *Nicotiana. benthamiana* enhanced host susceptibility to rice stripe virus (RSV) [29]. Moreover, overexpression of Psbp in *N. benthamiana* revealed that PsbP was involved in host defense response to geminivirus [39]. Similarly, Rodriguez-Herva and associates confirmed that silencing of PsbQ in tobacco compromised plant immune responses induced by *Pseudomonas syringae* pv tomato DC3000 [40]. In our research, PsbP and PsbQ were identified to be remarkably downregulated in response to MCMV, indicating that the virus may modulate the abundance of defense-related proteins from the chloroplast and benefit its infection.

### 3.2. Ribosomal Proteins Commonly Affected by MCMV Infection

Viruses depend on their host ribosomal machinery to synthesize viral proteins. Our comparative proteomic analysis revealed that most of the ribosome-related proteins were upregulated in response to MCMV infection. Thus, we speculated that the increase in the abundance of ribosomal proteins during MVMV infection might help host plants to survive viral infection. Further studies will be required to understand the mechanism of ribosomal proteins involved in viral translation and replication. Recently, Li and associates reported that knockdown of ribosomal proteins L18 (rpL18) reduced RSV accumulation in small brown planthopper through suppression of the interaction between rpL18 and RSV nucleocapsid protein [41]. Previous evidence also indicated that tobacco etch virus (TEV) P1 protein could specifically bind with host’s 60S ribosomal subunits and stimulate protein translation in vitro translation assays [42]. Conversely, it is worth noting that a plant NSP-interacting kinase mediates nuclear trafficking of ribosomal protein L10A (rpL10A), which enhances plant resistance to geminivirus infection [43]. Furthermore, ribosomal proteins rpL12 and rpL19 were also confirmed to be important factors in non-host disease resistance in *N. benthamiana* and Arabidopsis against bacterial pathogens [44]. Obviously, the programming of ribosome-related proteins might maintain a balance between plant immune response to virus and viral infection.

### 3.3. Stress Response-Related Proteins Regulated by MCMV Infection

Our comparative proteomic analysis found that defense- and stimulus-related DAPs were significantly modulated in abundance after MCMV infection. Several LOXs upon MCMV infection were showed to be differently regulated, suggesting a subtle response of LOXs after MCMV infection. Previous evidences had showed that the activities of LOX-derived oxylipins were induced during defense response against diverse pathogens [45]. As known, the monocot-specific 9-LOX was required to initiate an effective jasmonate-mediated defense response against *Fusarium verticillioides* in diverse maize tissues [46]. Also, the abundance of endogenous 9-LOX was highly induced after agro-infiltration with TMV-based construct in *N. benthamiana* [45]. Not only the expression change of diverse LOXs, in our study, the abundance of ribosome-inactivating protein 2 and HSP70 were also altered in response to MCMV infection. Homologous proteins of ribosome-inactivating protein from the genus *Phytolacca* were reported to mediate a broad-spectrum antipathogenic activities [47]. In addition, heat shock protein were revealed to act as one of the major chaperones and involved in plant immunity and stress responses [48]. For example, maize HSP70 is reported to involve ABA-induced tolerance to drought and heat stress combination [49].

### 3.4. Redox Regulation in Maize after MCMV Infection

The rapid ROS production, known as the oxidative burst, is the earliest defense response employed by plants [50]. ROS are the primary signals during plant defense response and interact with plant hormones, nitric oxide, mitogen-activated protein kinases (MAPKs), and many other signal compounds [51,52,53]. However, in host cells, highly reactive ROS can cause oxidative damage. Considering their toxicity and important role in signaling, the ROS level in cells must be tightly regulated. Thus, plants have evolved an enzymatic ROS scavenging system including different enzymes such as catalases (CAT), peroxidases (POD), and superoxide dismutases (SOD) to maintain a balance of endogenous ROS [50]. Previous studies on maize pathogens indicated that CAT activity was significantly increased in maize lines resistant to *Aspergillus flavus* infection and SOD activity was remarkably promoted on maize roots during *F. verticillioides* infection [54,55]. Huang and associates revealed that combined expression of APX and glutathione peroxidase contributed to maize defense response against *Curvularia lunata* by modulating the balance of ROS [56]. For viral infection, in SCMV-infected maize leaves, the proteomic level of polyamine oxidase (PAO) that catalyzes the production of H_2_O_2_ was upregulated, while the level of APX was strongly suppressed [26]. Consistent with these previous reports, in the present study we also found that 2-oxoglutarate dehydrogenase E1, a component that plays vital role in the generation of ROS, was increased in abundance while two scavenging enzymes, monooxygenase and isocitrate dehydrogenase, showed decreased abundance in MCMV-infected leaves. Obviously, our iTRAQ data suggested that maintaining redox dynamic balance in maize is required during MCMV infection.

### 3.5. ZmPDIL-1 Facilitates MCMV Infection

The family of protein disulfide isomerases (PDIs), which is highly conserved across eukaryotic species, is composed of several well-characterized proteins containing catalytic sites similar to those of thioredoxin [57]. PDIs have been shown to be involved not only in the folding of nascent polypeptides, but also in the formation of disulfide bonds in the endoplasmic reticulum [58]. Previous reports identified that PDIs inhibitor significantly reduced the replication of multiple human and animal viruses such as dengue virus, influenza A, and influenza B viruses [59,60]. In plants, the ER-resident PDIs are also known to have chaperone activities [61], and required for *N*-mediated resistance to TMV through accelerate the accumulation of induced receptor-like kinase during plant innate immunity [62]. In addition, a PDI-like protein HvPDIL5-1 derived from barley was confirmed to function as a susceptibility factor to bymoviruses infection [63]. Similarly, the iTRAQ analyses revealed that maize PDIL expression is upregulated in leaf tissues after SCMV infection, and knockdown of PDIL through brome mosaic virus-based VIGS can impair SCMV replication [26]. In the present study, we determined that ZmPDIL-1 was upregulated at both the protein and transcription levels after MCMV infection. Furthermore, the accumulation of MCMV in ZmPDIL-1-silenced maize plants was significantly decreased, suggesting that maize ZmPDIL-1 might be a common susceptibility factor to virus infection. However, it remains to be determined whether ZmPDIL-1 functions as a molecular chaperone for assisting correct folding of viral protein(s) or activating the host protein(s) involved in regulating virus infection.

### 3.6. ZmPrx5 Is a Positive Regulator of MCMV Infection

Peroxiredoxin protein (Prx) participates in the active oxygen scavenging through the oxidation of the conserved cysteine residue and reduction the ROS [64]. Several reports suggested that Prx contributed to propagation of human and animal viruses. Watanabe and associates found that knockdown of Peroxiredoxin 1 (Prx 1) by RNA interfering resulted in a significant reduction of measles Virus (MeV) replication [65]. Further study showed that Prx1 might serve as a host factor binding to MeV-NTAIL (the C-terminal region of the nucleoprotein) and positively regulated the transcription and replication of MeV [65]. Also, Deng and associates revealed that Prx1 could interacts with exosome component 5 and negatively regulate hepatitis B virus infection through RNA decay [66]. In plants, PRX genes have been characterized and mainly classified into four family members as 1-Cys Prx, 2-Cys Prx, Prx II, and Prx Q [67,68]. Kim and associates identified that overexpression of Arabidopsis 2-Cys Prx in transgenic tall fescue enhanced tolerance against methyl viologen and heat stresses [69]. Additionally, overexpression of a Prx Q homolog of *Gentiana triflora* in tobacco improved tolerance against fungi and enhanced anti-oxidation ability [70]. In the present study, our results indicated that silencing of ZmPrx5 through VIGS reduced the accumulation of MCMV in maize, indicating intracellular peroxiredoxin homeostasis may play a positive role in MCMV infection. However, the mechanisms whereby ZmPrx5 regulating MCMV propagation are still unknown. Our efforts are underway to identify viral protein(s) that might interaction with ZmPrx5.

## 4. Materials and Methods

### 4.1. Plant Growth and Virus Inoculation

The lowest two leaves on each 4-leaf stage maize cv. B73 plant were mechanically inoculated with crude extracts from MCMV-infected maize leaf tissues. The inoculated plants were grown in growth chambers with a 16:10 h (light: dark photoperiod) and 28:25 °C (day: night temperature cycle).

### 4.2. Sample Preparation

Leaves were collected at 11 days post inoculation (dpi) and stored at −80 °C until further use. For each treatment, two biological replicates with five individual plants each were used for the iTRAQ-based assay. The urea extraction method described by Sheoran et al. was used to extract total proteins from individual samples [71]. The concentrations of protein samples were measured using a Pierce™ Coomassie Plus (Bradford) Assay Kit (Thermo Fisher Scientific, Wilmington, NC, USA) and protein samples were kept at −80 °C for later analysis.

### 4.3. iTRAQ Labeling and Strong-Cation-Exchange (SCX) Fractionation

Total protein (100 μg) from each sample was mixed with Trypsin Gold (Promega, Madison, WI, USA) at a ratio of 30:1 (*v*/*v*) and incubated at 37 °C for 16 h. Thereafter, vacuum centrifugation was used to dry the released peptides, which then reconstituted in 0.5 M tetraethyl-ammonium bromide (TEAB). The 8-plex iTRAQ reagent (Applied Biosystems, Foster City, CA, USA) was used to further process the samples, according to the manufacturer’s protocol. One unit of iTRAQ reagent was mixed with 24 μL of isopropanol. The protein samples were labeled with the iTRAQ tags to achieve sample M1-113 tag, sample M2-115 tag, sample Mock1-118 tag, and sample Mock1-121 tag. After labeling, the samples were incubated at room temperature for 2 h, pooled, and then dried by vacuum centrifugation. Then, SCX chromatography was performed with a LC-20AB HPLC Pump system (Shimadzu, Kyoto, Japan) as described [72]. Briefly, the iTRAQ labeled peptide samples were individually mixed with 4 mL of buffer A (25 mM NaH_2_PO_4_ in 25% ACN, pH 2.7) and loaded onto a 4.6 × 250 mm Ultremex SCX column containing 5 μm particles (Phenomenex, Torrance, CA, USA). The peptides were eluted at a flow rate of 1 mL/min with a linear gradient of buffer B (25 mM NaH_2_PO_4_, 1 M KCl in 25% ACN, pH 2.7). The gradient was as followed: 5 min in 5% buffer B, 27 min in 5–60% buffer B, 2 min in 60–100% buffer B. The system was then maintained in 100% buffer B for 1 min. The eluted fractions were collected at 1 min intervals and monitored by measuring the absorbance at 214 nm. The fractions obtained were pooled into 20 fractions, subjected to desalting using a Strata X C18 column, and dried under a vacuum.

### 4.4. Liquid Chromatography-Tandem Mass Spectrometry (LC-MS/MS)

Peptides from each fraction was resuspended in buffer A (2% ACN, 0.1% FA) and centrifuged at 20,000× *g* for 10 min. The final concentration of peptide was about 0.5 μg/μL on average. A 10 μL sample was loaded onto a LC-20AD nano-HPLC column (Shimadzu, Kyoto, Japan) using an autosampler into a 2 cm C18 trap column at 8 μL/min flow rate. Then the peptides were eluted onto a 10 cm analytical C18 column (inner diameter 75 μm) packed in-house, at a flow rate of 1 mL/min with a linear gradient of buffer B (95% ACN, 0.1% FA). The gradient was as followed: 5 min in 5% buffer B, 35 min in 5–35% buffer B, followed by a 5 min linear gradient to 60% and 2 min linear gradient to 80%, maintained at 80% B for 4 min, and finally a return to 5% in 1 min. The MS analysis was performed using a a TripleTOF 5600 System (AB SCIEX, Concord, ON). Intact peptides were detected in the Orbitrap at a resolution of 30,000 and were selected for MS/MS using high-energy collision dissociation (HCD) operating mode with a normalized collision energy setting of 27.0, and ion fragments were detected in the Orbitrap at a resolution of 19,000. For MS1 scans, the m/z scan range was 350 to 2,000 Da. For MS2 scans, the m/z scan range was 100 to 1,800 Da.

### 4.5. Proteomics Data Analysis

Protein identification was performed by searching the Mascot database (version 2.3.02, Matrix Science, London, UK). The peptides’ charge states were set to +2 and +3. Specifically, in Mascot, an automatic decoy database search was performed, by selecting the decoy checkbox. This generates a random sequence of the database that is tested for the raw spectra and used as the real database. To decrease the likelihood of false positive peptide identification, only those peptides with a 95% confidence interval greater than ‘identity’, as set by a Mascot probability analysis, were counted as identified. Each confidently identified protein involved at least one unique peptide. To quantify the proteins, each protein was required to contain at least two unique spectra. IQuant was used for protein quantification with VSN normalization. For each protein, IQuant provided a significance evaluation that was corrected for multiple hypothesis testing by the Benjamini–Hochberg method. As described previously [73], proteins were considered as significantly differently abundant if they had *p*-values ≤ 0.05 and mean fold change ≥ 1.2 or ≤ 0.83.

Proteome Discoverer 1.2 software (version PD 1.2, Thermo, SanJose, CA) was used to convert the Orbitrap-generated raw data into MGF files. The Mascot search engine was used to identify the proteins using a database containing the B73 maize genome sequences (https://www.maizegdb.org; B73 RefGen v3), the non-redundant protein database (NR; NCBI) and Maize SwissProt database. All raw MS files are publicly available for download from the proteomic data repository webserver, iProX (http://www.iprox.org/) with the project number IPX0001435000.

### 4.6. Annotation of the Proteins

The identified proteins were functionally annotated using gene ontology (GO) analysis in the Blast2GO program. To classify and group the identified proteins, the Kyoto Encyclopedia of Genes and Genomes (KEGG) (http://www.genome.jp/kegg/) and Clusters of Orthologous Groups (COG) databases (http://www.ncbi.nlm.nih.gov/COG/) were used.

### 4.7. Enzyme-Linked Immunosorbent Assay (ELISA)

ELISA assays were conducted as described previously [73]. For samples co-infected with MCMV and SCMV, two duplicated plates were used, and one was incubated with a MCMV-specific monoclonal antibody [73] and the other was incubated with a SCMV-specific monoclonal antibody [74].

### 4.8. Total RNA Extraction and qRT-PCR Assay

Total RNA from individual samples was isolate using the TRIzol Reagent (Invitrogen, Carlsbad, CA, USA). A nano-drop spectrophotometer (Thermo Fisher Scientific) was used to monitor the concentration and quality of the total RNA samples, followed by agarose gel electrophoresis. For each RNA sample, the concentration was adjusted to 1 μg/μL. Reverse transcription (RT) was then performed using 1 μg of total RNA in each 20 μL RT reaction using the ReverTra Ace qPCR RT Kit (TOYOBO, Osaka, Japan). To confirm the results from the iTRAQ-based proteomic analyses, the RNA transcript levels of 27 randomly selected differentially expressed proteins (DAPs) were determined by qPCR using the LightCycler 480@ SYBR Green I Master (Roche Applied Science, Basel, Switzerland). For each gene, three technical replicates were used during qPCR to calculate the gene’s relative expression. Gene expression was normalized against the expression level of maize endogenous elongation factor-1 A gene (EF-1A). The primers used for PCR or qRT-PCR are listed in Table S7. Cucumber mosaic virus-based virus-induced gene silencing (CMV-VIGS) plasmid construction and maize inoculation

CMV-VIGS in maize was conducted as described by Wang et al. with minor modifications [75]. Briefly, construct pCMV101, pCMV301, or the pCMV201 derivatives were transformed into the C58C1 strain of *Agrobacterium tumefaciens*. Cultures of agrobacterium containing pCMV101, pCMV301, and one of the pCMV201 derivatives were co-infiltrated into Nicotiana benthamiana leaves. At 4 dpi, the infiltrated *N. benthamiana* leaves were harvested, ground in phosphate buffer (0.1 M, pH 7.0), and subjected to centrifugation at 3,000× *g* at 4 °C for 3 min. The vascular puncture inoculation (VPI) method was used to inoculate maize seeds with the recovered supernatants. Seeds of maize cv. B73 were soaked for 30 min in tap water at room temperature. Virus-containing supernatant was pipetted onto the surface of a seed alongside the embryo (15 μL per kernel) and a vibrating engraving tool was used to inoculate the supernatant into the underlying vascular bundles. The control comprised a vector carrying a fragment (254 bp) of the green florescent protein (GFP) gene. The inoculated seeds were incubated for 2 days in the dark at 25 °C and then transplanted into pots containing soil. The resulting plants were cultured in a growth chamber with a 16:8 h light and dark photoperiod set at 28:25 °C (day:night). For each treatment, 30–40 seeds were inoculated.

### 4.9. Measurements of Photosynthetic Parameters

The effects of MCMV on maize photosynthesis were determined with Imaging PAM (IMAG-MAXI; Heinz Walz, Effeltrich, Germany) as described previously [2].

## 5. Conclusions

In the present study, we performed iTRAQ-based quantitative proteomic analysis to investigate the responses of maize to MCMV infection. In the paired comparisons of MCMV infection and mock-inoculated samples, a total of 972 proteins were identified as DAPs with significant changes. The differential abundance of the majority DAPs involved in photosynthetic metabolism and ribosome-related pathways, and those related to stress responses and redox regulation were remarkably induced during MCMV infection. Furthermore, the roles of two candidates, ZmPDIL-1 and ZmPrx5, were investigated using CMV-induced gene silencing approach. Our results indicated that combining comparative proteomic analyses and virus-induced gene silencing can assist in identifying host proteins modulating MCMV infection and might contribute to provide guidelines for designing antiviral strategies.

## Supplementary Materials

Supplementary materials can be found at https://www.mdpi.com/1422-0067/21/1/35/s1.

## Abbreviations

MCMV	chlorotic mottle virus
CMV	cucumber mosaic virus
SCMV	sugarcane mosaic virus
MDMV	maize dwarf mosaic virus
Mev	measles Virus
sgRNAs	subgenomic RNAs
LOP	lipoxygenase
ROS	reactive oxygen species
HSP	heat shock protein
CAT	catalases
POD	peroxidases
SOD	superoxide dismutases
PDI	protein disulfide isomerases
Prx	peroxiredoxin protein
