# Supplementary material for: Proteomic Changes during MCMV Infection Revealed by iTRAQ Quantitative Proteomic Analysis in Maize
Source: Int J Mol Sci. 2019 Dec 19;21(1):35. doi: 10.3390/ijms21010035 (PMC6981863; doi:10.3390/ijms21010035)
Supplement: Supplementary file 1 [file ijms-21-00035-s001.zip › ijms-652724-publish supplementary/Table S7-Primers used for qRT-PCR.doc]

| **Table S7** Primers used for qRT-PCR analysis and construction VIGS vector | | |
| --- | --- | --- |
| **Primers used for qRT-PCR analysis** | |  |
| **Protein ID** | **Primer sequence (5'→3')** |  |
| GRMZM6G729818_P01-F | caatcagagccagtatcctcgcatt |  |
| GRMZM6G729818_P01-R | ttgccctgagccaataagatgtcgag |  |
| GRMZM2G030523_P02-F | aacgacgacatcatcaccataaaggc |  |
| GRMZM2G030523_P02-R | ataaactcagaagaaggcatgcggac |  |
| GRMZM2G330635_P01-F | gcgacgctctcctacttcgaca |  |
| GRMZM2G330635_P01-R | cttggccacgaaccccggga |  |
| AC218900.3_FGP001-F | aagagaggcgccaaggtgaaga |  |
| AC218900.3_FGP001-R | atttcacctcgaggatctcgtccaa |  |
| GRMZM2G079538_P03-F | tcccaagatgcaagcaccaaagc |  |
| GRMZM2G079538_P03-R | cagcattgcaaaggtgttctgagaa |  |
| GRMZM2G018417_P01-F | atcaacacctttggttgagctcgc |  |
| GRMZM2G018417_P01-R | cgtaagatgccttcaaagtctcccc |  |
| GRMZM5G824920_P01-F | tacctcaatgtcaccaacgtgccg |  |
| GRMZM5G824920_P01-R | tacagctcgtagatgtaggtcggcac |  |
| GRMZM2G153823_P01-F | aagcagaacggtacgaggaaatgg |  |
| GRMZM2G153823_P01-R | ggactcctccttatgctcgatgg |  |
| GRMZM2G145496_P01-F | caagatatacggcgaccagctcg |  |
| GRMZM2G145496_P01-R | acactaacctttttcttgtccggcc |  |
| GRMZM2G091481_P01-F | GAGGCTGATGGCATTGTGGATTACTTG |  |
| GRMZM2G091481_P01-R | CTTCAGCAGCCTGACCAATGGCCGTT |  |
| GRMZM5G804323_P01-F | gtggcgagggtgaagacgtcg |  |
| GRMZM5G804323_P01-R | aagagcttgccggtgagctcc |  |
| GRMZM2G162529_P01-F | cgacaagccggtggtgattggc |  |
| GRMZM2G162529_P01-R | ccgttcctgtccagggaatggtag |  |
| GRMZM2G181258_P01-F | atggccctggagacgtgcttca |  |
| GRMZM2G181258_P01-R | ggcgttcttggactggcagacg |  |
| GRMZM2G146292_P04-F | agaaagacttgaagaactggcggtgg |  |
| GRMZM2G146292_P04-R | ctgagaaagatctgcgcattttgcag |  |
| GRMZM2G036921_P01-F | CGACTCTGTTATCTGTGTAGCTGTGAATG |  |
| GRMZM2G036921_P01-R | TCAGATCTGGTCCAAGATCACCTCCGC |  |
| GRMZM2G462613_P01-F | ctatccacgcatcacgctctcag |  |
| GRMZM2G462613_P01-R | atctcctcaccgtgtggatcct |  |
| **Primers used for construction of VIGS vector (5’→3’)** | |  |
| CMV-ZmPDIL-1-F | AAATGTCCGAGTCTGAGGTACCTGGTGTGGGCACTGCAAGAAACTTG |  |
| CMV-ZmPDIL-1-R | ATTAGGAAGGGGAGGTTCTAGATGGTGACTTGATCTCCTTGGACGCA |  |
| CMV-Prx5-F | AAATGTCCGAGTCTGAGGTACCCCAAGTTCTCTACCACGCCTCTCAAG |  |
| CMV-Prx5-R | ATTAGGAAGGGGAGGTTCTAGACTCCCATCAAAGTCACCATAGAACTCAA |  |
